# Supplementary material for: Risk of cutaneous squamous cell carcinoma due to occupational exposure to solar ultraviolet radiation: Protocol for a systematic review and meta-analysis
Source: PLoS One. 2023 Mar 3;18(3):e0282664. doi: 10.1371/journal.pone.0282664 (PMC9983864; doi:10.1371/journal.pone.0282664)
Supplement: Attachment — Submitted filename: Response to Reviewers PONE-D-22-18577_ 25 Jan 23.docx [file pone.0282664.s002.docx]

Dear Editor,

Thank you for your letter and the valuable review of our manuscript (PONE-D-22-18577). My co-authors and I reviewed the comments and we believe that our manuscript has been improved as a result of the review process. Please find attached to this letter (i) the clean revised manuscript and (ii) the revised manuscript with tracked-changes. Thank you for providing us with the opportunity to submit an improved version of our protocol to Plos One.

| **Reviewer #1** | **Authors** |
| --- | --- |
| The protocol that is presented is to conduct a systematic review (and possibly a meta-analysis if there are sufficient quality articles) on the relationship between cSCC and occupational solar UVR exposure. The protocol is very generalized and needs additional detail - bearing in mind the thinking of a protocol is for someone else to follow the 'recipe' to repeat the study in the future. | Thank you very much for the time taken to revise our protocol and your comments. We have detailed the protocol in accordance, see below in more detail.  Please see lines 243-251, page 5; line 437-454, page 7; line 551-623, page 8. |
| Please provide more detail on these aspects: by sex, occupation, country or region, latitude, the number of years exposed (number of years as outdoor workers), exposure levels, and the adoption of solar UVR protections. | We adjusted as follows “ (…) sex (male/female), occupation (type of occupation as described in the study), country, region (WHO region), latitude (from the city where data was collected), the number of years exposed (number of years as outdoor workers), non-occupational exposure, exposure levels (medium or moderate, high), and the adoption of solar UVR protections.”  Please see lines 553-557, page 8. |
| Some detail is given about 1 SED per day, but how will latitude be investigated, for example. Will countries be categorized or analyzed as individual countries, etc. | We will search the latitude of the city where data was collected to perform a sensitivity analysis taking that into consideration. Countries will be used as individual countries, e.g., if there is more than one risk estimate per country, we will use both, and we will also categorize countries per WHO world region in a sensitivity analysis.  Please see lines 557-621, page 8. |
| How will occupation be assessed - by category e.g., water and solar UVR exposure, snow and solar UVR exposure, or just by type of occupation? | Yes, occupation and category will be assessed by its type as described in the original study.  Please see line 554, page 8. |
| How will personal, non-work time exposure be taken into consideration in the study of cSCC and occupational solar UVR exposure? In order to assess causation in the workplace. I would expect to see how this will be considered in the protocol given the ubiquitous nature of sun exposure. | We will extract data on non-occupational exposure as provided by the original study.  Please see line 554, page 8. |
| Has another review similar to the one proposed in this protocol be done before? Or similar? Please mention in the introduction. | We thank Reviewer 1 for their comment.  Please see lines 243-251, page 5. |
| Why was the review process started a year ago and the protocol is still under review with a journal? Has the review work not begun yet? It should have waited until the protocol was peer reviewed, in addition to registration on PROSPERO. | We submitted it to Plos ONE on the 4^th^ of July 2022 where we were waiting for the review process. We are currently pausing the process while we do not have the publication of the protocol to start the actual review. |
| Articles from all languages will be included in the systematic review but it is not stated whether there are people in the team who speak all languages around the world, or how this process will be managed. | “There will be no date or language restrictions, authors speak Dutch, English, German, Hungarian, Italian, Portuguese, and Spanish. Any other eligible paper published in another language, will be translated using an online platform, and validated by a colleague from the nine academic institutions where the authors are affiliated with.”  Please see line 450-454, page 7. |
| It would be helpful to know the time frame of the proposed work. | We will start the search at the three databases as soon as the protocol is accepted for publication. We anticipate that we will need one year until the conclusion of the full systematic review and meta-analysis. We added information about it.  “The searches will start upon acceptance for publication of the present protocol and will be re-run just before the final analyses and further studies retrieved for inclusion.”  Please see line 453-455, page 7. |
| **Reviewer #2:** | **Authors** |
| It is of importance to critically assess the increased risk of developing cutaneous SCC in outdoor workers due to exposure to solar UV radiation. Such an investigation is difficult to undertake due to the many variables involved but is worthwhile not only in terms of personal health but also for drawing up relevant legislation in the future for worker protection. The proposed systematic review and meta-analysis described in this article will, hopefully, provide useful information to add to that available in various recent published reviews and papers. | Dear Reviewer 2, thank you very much for the time taken to revise our protocol and your comments. The significant changes preformed are in lines 243-251, page 5; line 437-454, page 7; line 551-623, page 8. |
| Search terms used to identify relevant articles in the academic databases are not given. | “Search terms will follow the PECO criteria outline above including a combination of workplace, employment, occupational exposure, exposed worker, outdoor workers, workers, occupation, outdoor job, outside occupation, outside work, nonionizing radiation, sunlight, ultraviolet rays, solar radiation, ultraviolet radiation, UV rays, squamous cell carcinoma, skin cancer, and skin malignancies.”  Please see line 437-441, page 6. |
| The Methods were brief. More detail is required as follows. First the phototype of the potential participants is not mentioned which is of importance as those with darkly pigmented skin would be much less likely than those with fair skin to develop SCC through occupational solar UV radiation exposure. | We will extract data reporting the phototype of the participants and we will use the most adjusted estimate provided in the original study. As far as we know, in these types of studies, it is frequent to find risk estimates adjusted for sex, age and phototype.  “We will extract the estimate with and without adjustment and we will use the most adjusted one to pool our estimates. We expect to find risk estimates adjusting for sex, age and phototype.”  Please see line 621-623, page 9. |
| Secondly the exposure was defined as 1 SED/day. Is this every day throughout the year or only in the summer months where relevant? In addition the dose of UV radiation can be assessed in terms of personal dosimeters, ambient radiation, UV Index or questionnaires relating to work practices/past sunburns. Would all these methods be acceptable? | We believe most of the studies eligible for inclusion will have their exposure reported using indirect measures (self-reported, from their employment, assessed by a health and safety technician). For direct measurements we will extract data from published papers with specific information about how they measured the exposure (type of dosimeter, the location of the dosimeter according to the job task, the time of the year and the weather when the measurements were done). Differences in exposure assessment will be assessed before any decision about meta-analysis; exposure assessment will be rated in Risk of Bias assessments.  Please see line 404-406, page 6. |
| Thirdly the Outcome is stated as being “medical diagnosis” but this can vary from examination by pathology of a biopsy to that suspected by a General Practioner. Would all these be acceptable? | We have clarified now that we will just accept histologically confirmed diagnosis.  “The primary outcome is cSCC occurrence based on a histologically confirmed medical diagnosis (including registry data). Secondary outcomes are SCC in situ (also referred to as Bowen’s disease) and AK based on a histologically confirmed medical diagnosis (including registry data).”  Please see line 410-412, page 6. |
| There are a number of small errors throughout such as the superscript 9 in the title which does not relate to an author | We thank Reviewer 2 for highlighting our small errors; we have corrected the affiliation accordingly.  Please see page 1. |
| 1.8 M cases of cSCC out of 7.7 M MNSCs is the same as 20% so this does not need to be stated twice (lines 6-8, Introduction), the sentence beginning “About the number…..” requires rewriting (beginning of Second paragraph, Introduction), and there are lot of unnecessary hyphens throughout. | We have improved the text in the Introduction.  Please see page 3. |
| **Editor** | **Authors** |
| Please add details on the research string to be used in Medline through PubMed, EMBASE, Scopus. | We thank the Editor for their comment. We have added the terms used to construct the search strings.  “Search terms will follow the above reported PECO criteria outlined, and will include: the following factors: workplace, employment, occupational exposure, exposed worker, outdoor workers, workers, occupation, outdoor job, outside occupation, outside work, nonionizing radiation, sunlight, ultraviolet rays, solar radiation, ultraviolet radiation, UV rays, squamous cell carcinoma, skin cancer, and skin malignancies. The search was designed for PubMed and using medical subject headings (MeSH) as well as title and abstract forms (please see Supplementary material). The search will be translated to Scopus and EMBASE following the same structure..”  Please see line 437-444, page 6 and Supplementary material. |
| Do you plan to contact study authors’ in case you cannot retrieve data from the published reports? If so, please specify it. | We have added more information on this aspect of search.  “Contacting authors to request data on specific studies will – if necessary – be done by sending an email to the corresponding author and a follow-up email after two weeks. In case that the corresponding author does not answer after one month, the study will be excluded and document in an appendix file.”  Please see line 447-450, page 7. |
| You declared that you will present the results of the meta-analysis separately for case-control and cohort studies. Being aware of the potential bias of case-control studies, I was wondering why you will not plan to pool together both study types by providing a stratified forest-plot. | We do agree with you regarding pooling data from cohort studies and case-controls studies. We will do as you suggest and perform a pooled analysis stratified by type of study. The estimates used in the forest plot will be the most adjusted ones provided by the studies, if they are the same adjustments (usually, age, sex and phototype).  “If possible, meta-analyses will pool stratified estimates from cohort and case-control studies using random-effects model in RevMan 5.3.”  Please see line 550-552, page 8. |
| I cannot understand the sentence “If data estimates from the same study design are available for more than two studies, […]”. What do you mean? For example, the same study reporting separately results by sex, or by age-groups, without providing the overall estimate? Please clarify. | As we were planning to have separate analyses by study type, we were advocating that pooled analysis, per risk factor, would be done for more than two separate papers in case-controls and cohort studies. We will then perform sensitivity analyses.  “If more than two risk estimates are available without substantial heterogeneity (i.e. I2 <70%) we will perform secondary analysis by sex (male/female), occupation (type of occupation as described in the study) country, region (WHO region), latitude (from the city where data was collected), number of years exposed (number of years as outdoor workers), non-occupational exposure, exposure levels (medium or moderate, high), and the adoption of solar UVR protections.”  Please see line 552-557, page 8. |
| Please add more details on the statistical analysis plan; i.e., which model, fixed- or random-effects, which estimator of between-study heterogeneity, any sensitivity analysis, or meta-regression. | We have added information regarding the model to be used. “If possible, meta-analyses will pool stratified estimates from cohort and case-control studies using random-effects model in RevMan 5.3.” Of course, we will explore any (statistical) heterogeneity and secondary analysis will be performed having that into consideration.  Please see line 550-557, page 8. |

Best regards,

Priv.-Doz. Dr. rer. nat. habil Marc Wittlich

Institute for Occupational Safety and Health of the German Social Accident Insurance

Sankt Augustin, Germany

Email: marc.wittlich@dguv.de
