# Supplementary material for: Risk of cutaneous squamous cell carcinoma due to occupational exposure to solar ultraviolet radiation: Protocol for a systematic review and meta-analysis
Source: PLoS One. 2023 Mar 3;18(3):e0282664. doi: 10.1371/journal.pone.0282664 (PMC9983864; doi:10.1371/journal.pone.0282664)
Supplement: Attachment — Submitted filename: 2nd Response to Reviewers PONE-D-22-18577_ 09 Feb 23.docx [file pone.0282664.s003.docx]

Dear Editor,

Thank you for your letter and the valuable review of our manuscript (PONE-D-22-18577). My co-authors and I reviewed the comments and we believe that our manuscript has been improved as a result of the review process. Please find attached to this letter (i) the clean revised manuscript and (ii) the revised manuscript with tracked-changes. Thank you for providing us with the opportunity to submit an improved version of our protocol to Plos One.

| **Reviewer #2:** | **Authors** |
| --- | --- |
| Although the revised manuscript is improved compared with the original submission, there remain a number of aspects for consideration by the authors, as follows | Dear Reviewer 2, thank you very much for the time taken to revise our protocol and your comments. The significant changes preformed are in lines 38, 39, 44, 177, and 252. |
| Line 36 and elsewhere – why are there comments in the right hand margin? | For full transparency we have used track changes, therefore you were able to see all the comments in the right hand margin, but we have also submitted a clean version of the manuscript and we will do so this time as well. |
| Line 38. How many readers of this article will know the current occupational exposure limit for artificial UVR? The actual limit should be stated especially as artificial UVR is not mentioned in the text of the article. | We have added the information about the theoretical minimum risk exposure level as ≥1.0 SED/day.  Please see line 38, page 1. |
| Line 39-40. It is unclear what “continuous and cumulative patterns of sun exposure” means. Suggest replacing this by “chronic and intermittent sun exposure”. | Thank you, we have replaced it.  Please see line 39, page 1. |
| Line 42. Add age and phototype to this list. | Thank you, we have added it  Please see line 44, page 2. |
| Line 50. What does “potentially expected globally” mean? | Potentially expected globally, means that every year there is an expectation or it is expected that this type of cancer will be highly diagnosed. |
| Line 153. The statement that that “The exposure studied is defined as 1 SED per day which can be achieved by spending more than one hour of the working day by outdoor workers during daylight hours….” cannot be valid for those working outdoors in the winter months, say at latitudes above 45 degrees. See, for example, Soueid et al Br J Dermatol showing that the median daily measured personal UVR dose for an outdoor worker is 1.3 SED in winter at a latitude of 41 degrees north, and would obviously be less at higher latitudes. | Dear Reviewer, thank you for the thought-provoking comment. You are right, we also see differences in latitudes if we compare. We don’t think it is feasible to have different metrics for different latitudes. Therefore, we have to set a limit to streamline our exposure metrics and this reference from Wittlich 2022 seemed to be achievable. |
| Line 156. Is latitude of residence or ambient UVR levels excluded? | Yes. We will ensure that on data extraction, we have added this information.  Please see line 159, page 6. |
| Line 161. It is not normal medical practice, at least in the UK, to biopsy Bowen’s or AK lesions. Relying on biopsy results for such lesions would therefore not give a valid picture of their frequency in outdoor workers. | Thank you for the explanation, and that is the reason why we consider AK and Bowen’s disease as secondary outcomes. They might be reported on recent studies. |
| Line 176. Why are Bowen’s and AK not included as search terms? | Thank you for the suggestion, we will look into it again, but in our preliminary searches, we found that these two terms were very limiting in terms on number of hits retrieved. |
| Line 232. Add body site of lesions (whether on a frequently UV-exposed site or not). | We have added it, and age and phototype according to your previous comment.  Please see line 233, page 8. |

Best regards,

Priv.-Doz. Dr. rer. nat. habil Marc Wittlich

Institute for Occupational Safety and Health of the German Social Accident Insurance

Sankt Augustin, Germany

Email: marc.wittlich@dguv.de
